# Concurrent aspergillosis and cystic pulmonary metastases in a patient with tongue squamous cell carcinoma

**DOI:** 10.1515/med-2022-0527

**Published:** 2022-07-20

**Authors:** Hung-Chieh Su, Che-Chi Liao, Chieh-Lung Chen, Wei Chih Liao, Wen-Chien Cheng

**Affiliations:** Department of Internal Medicine, China Medical University Hospital, Taichung, Taiwan; Department of Pathology, China Medical University Hospital, Taichung, Taiwan; Department of Internal Medicine, Division of Pulmonary and Critical Care Medicine, China Medical University Hospital, Taichung City 40402, Taiwan; Department of Internal Medicine, Hyperbaric Oxygen Therapy Center, China Medical University Hospital, Taichung, Taiwan; Department of Internal Medicine, Division of Pulmonary and Critical Care Medicine, China Medical University Hospital, No. 2, Yude Road, North District Taichung City 40402, Taiwan; School of Medicine, China Medical University, Taichung, Taiwan

**Keywords:** aspergillosis, cystic pulmonary metastasis, squamous cell carcinoma

## Abstract

Pulmonary *Aspergillus* infection may have a variety of manifestations depending on the patients’ immunity status and pre-existing lung conditions. Radiographically, aspergilloma, which is usually associated with noninvasive *Aspergillus fumigatus* conidia, may feature a characteristic mass in a cavity commonly located in the upper lobes of the lung. It is typically encountered upon pre-existing lung damage. Here we report *Aspergillus* growing in a pulmonary metastatic cavity in a 47-year-old male worker with a history of tongue cancer after a radical operation with neck dissection and concurrent chemotherapy in 2014. Chest radiography and computed tomography showed a cavitary lesion with a ball-in-hole lesion in the right upper lobe (RUL) and two cystic lesions within the bilateral upper lung field. Endobronchial ultrasound-guided transbronchial biopsy (EBUS-TBB) from the RUL anterior segmental bronchus (RB3) revealed the presence of *Aspergillus* conidia and squamous cell carcinoma. Wedge resection of the cystic lesion within the left upper lobe confirmed the diagnosis of metastatic squamous cell carcinoma. This is a rare case of aspergillosis within cavities of pulmonary metastases in a patient who was diagnosed with tongue squamous cell carcinoma. The conclusive distinction between neoplasm and fungal infection is difficult to achieve by radiography, and a pathological biopsy by EBUS-TBB is necessary to aid diagnosis. Clinicians should be aware of such an atypical presentation of metastases coexisting with *Aspergillus* infection.

## 1 Introduction

Pulmonary *Aspergillus* infection may have a variety of manifestations depending on the patients’ immunity status that is associated with a disorder of certain subpopulations of cells of the immune system [1] and any pre-existing lung conditions. These pre-existing conditions are not only predilection for the development of aspergillosis, but also many other conditions, always with a decrease in immune function, which are very common in leukemia [2]. The disease spectrum ranges from hypersensitivity pneumonitis and allergic bronchopulmonary aspergillosis to aspergilloma and even invasive aspergillosis. Typically, aspergilloma is a type of fungus ball that is usually related to noninvasive *Aspergillus fumigatus* conidia. Aspergilloma is typically observed growing within a pre-existing lung cavity or cyst as with prior tuberculosis, sarcoidosis, pneumatoceles, pulmonary sequestration or bronchogenic cyst [3]. Cystic lesions are defined as spherical parenchymal lucencies bordered by a thin wall of less than 2 µm (<2 mm) in thickness and a well-defined interface with the normal lung [4]. The differential diagnosis of focal or multifocal cystic lesions includes metastasizing neoplasms, infections, and traumatic injury. Although cavitary lung lesions are well-characterized findings in metastatic tumors, cystic lung metastases are rare. Squamous cell carcinomas are regarded as the most common type of cavitary metastasis observed radiographically, and they account for 69% of all cavitary metastases [5]. The coexistence of pulmonary aspergillosis and bronchogenic carcinoma has also been reported previously [6,7,8]. However, all of the aforementioned cases were primary lung cancer cases. Herein we report a rare case of aspergillosis within cavities of pulmonary metastases in a patient who was diagnosed with tongue squamous cell carcinoma.

## 2 Case report

**Figure 1:**
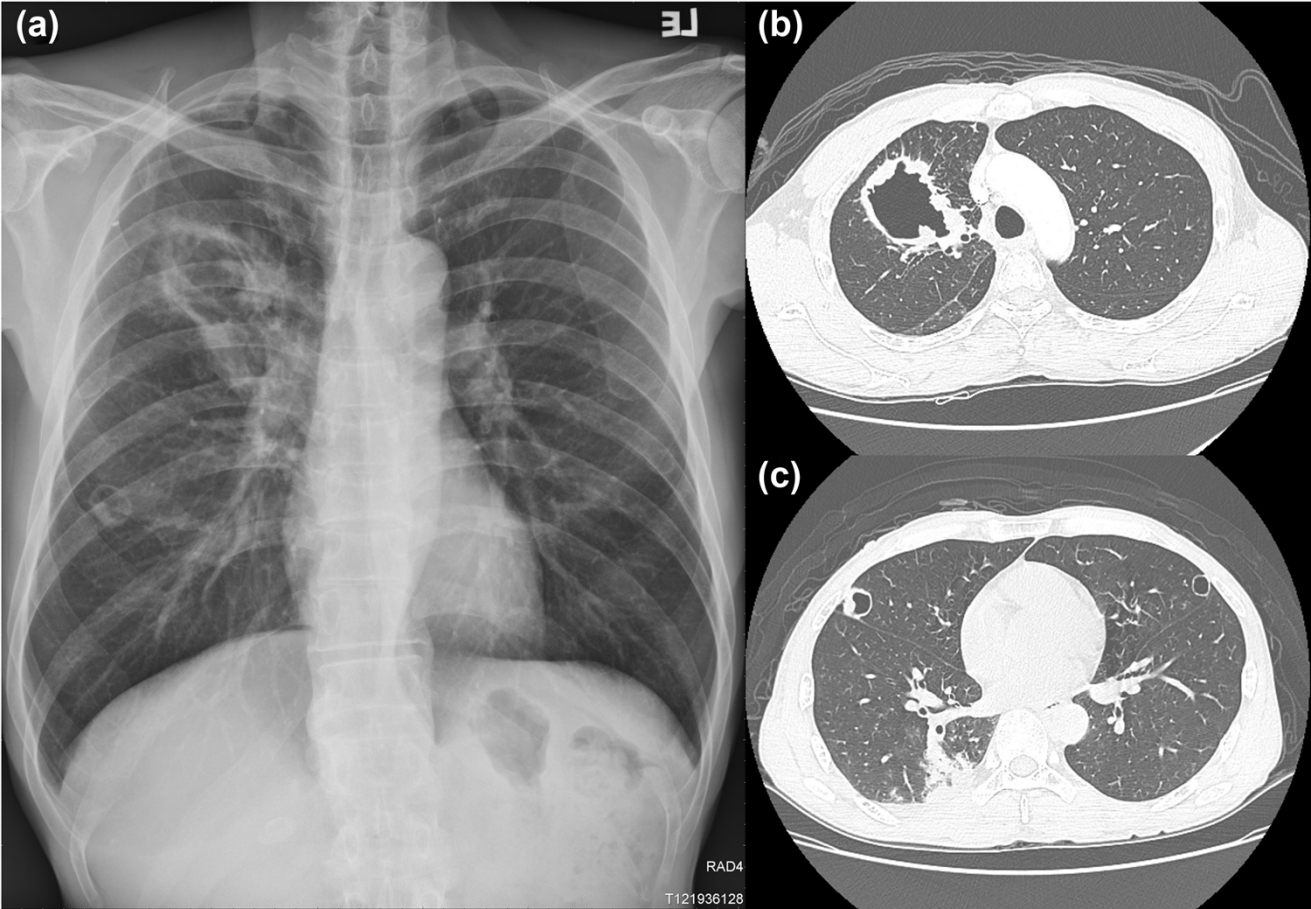
Chest radiography and computed tomography upon initial presentation: (a) RUL cavitary lesion and two cystic lesions within the bilateral upper lung field and (b and c) a cavity with a ball-in-hole lesion in RUL and two small cystic lesions in the bilateral upper lobes.

**Figure 2:**
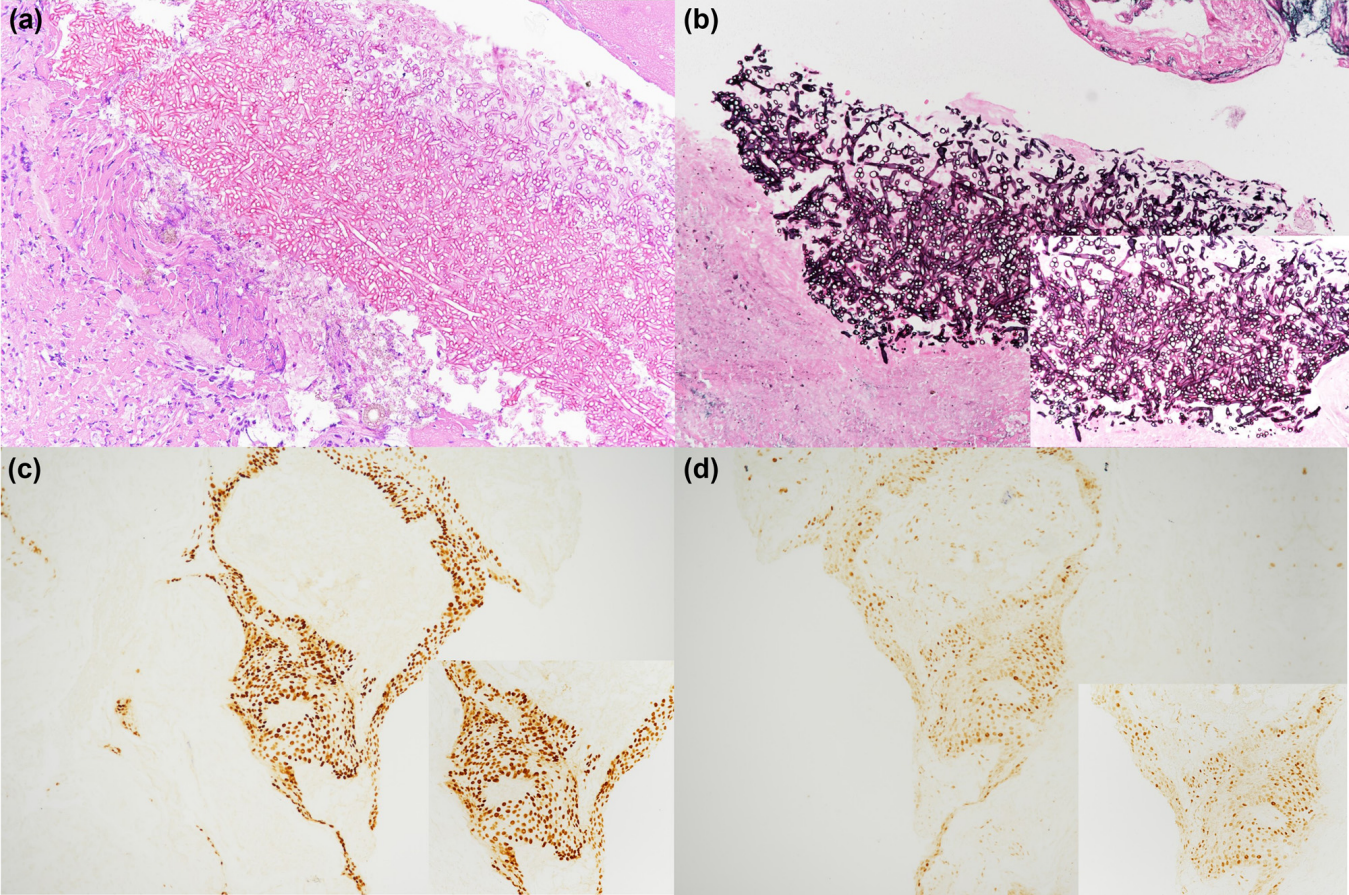
Pathology of metastatic squamous cell carcinoma coexisting with *Aspergillus fumigatus*: (a) clumps of septated fungal hyphae in hematoxylin and eosin stain. (H&E, 200×), (b) *Aspergillus fumigatus* in Grocott’s methenamine silver stain. (GMS, 200×); inset of a high-power microphotograph of GMS staining (GMS, 400×), (c) polygonal tumor cells arranged in solid nests and immunohistochemical study reveals positivity for p40 (IHC, p40, 100×); inset of a 200× microphotograph of p40 immunostaining (IHC, p40, 200×), and (d) polygonal tumor cells arranged in solid nests and immunohistochemical positivity for p53 (IHC, p53, 100×); inset of a 200× microphotograph of the p53 staining (IHC, p53, 200×).

**Figure 3:**
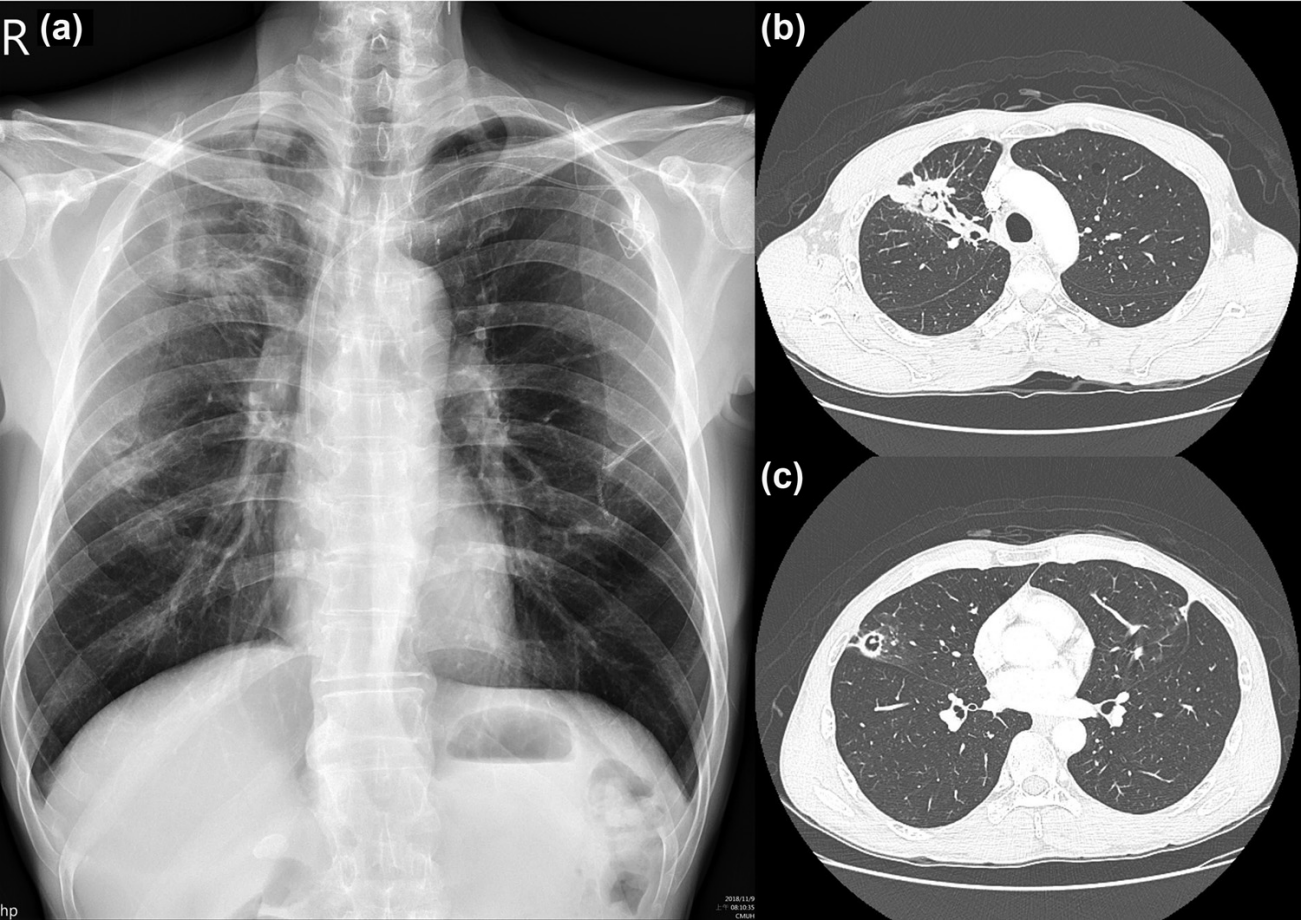
(a–c) Chest radiography and computed tomography following voriconazole administration and subsequent chemotherapy: regression of the pulmonary lesions on follow-up.


A 47-year-old male worker with a history of tongue cancer after radical operation with neck dissection and concurrent chemotherapy in 2014 came to our hospital presenting with right anterior chest pain and hemoptysis that had lasted for 1 month. No immunosuppressive medical history was shown for nearly 5 years before his first clinic visit and hospitalization, and after his last chemotherapy in 2014 for tongue squamous cell carcinoma. Moreover, his fasting blood sugar levels were within normal limit during every clinic visits. He denied having fever, dyspnea, dysphagia or abdominal pain. Chest radiography showed a right upper lobe (RUL) cavitary lesion and two cystic lesions within the bilateral upper lung field (Figure 1a). Chest computed tomography revealed a cavity with a ball-in-hole lesion in the RUL and two small cystic lesions in the bilateral upper lobes (Figure 1b and c). The first endobronchial ultrasound-guided transbronchial biopsy (EBUS-TBB) from the RUL anterior segmental bronchus (RB3) revealed the presence of *Aspergillus* conidia (Figure 2a and b). *Aspergillus fumigatus* was isolated from the bronchoalveolar lavage culture, and a serum *Aspergillus* galactomannan antigen test was found to be positive. Moreover, the histopathological examination of the biopsy also revealed the presence of squamous cell carcinoma (through immunohistochemical positivity for p40 and p53 and negativity for TTF-1), which was concurrent with aspergillosis (Figure 2c and d). The patient also received diagnostic wedge resection for the cystic lesion of the left upper lobe to differentiate between the fungal infection and the malignancy. Histopathology confirmed the diagnosis of metastatic squamous cell carcinoma based on clinical history and cell morphology. A final diagnosis of metastatic squamous cell carcinoma concurrent with *Aspergillus fumigatus* in the lung was made. Empirical voriconazole therapy for pulmonary aspergillosis was administered for nearly 2 months. Following voriconazole treatment, the serum *Aspergillus* galactomannan antigen test was negative, and no evidence of pulmonary aspergillosis in sequential bronchoalveolar lavage was found. Subsequent chemotherapy with cisplatin and docetaxel was undertaken for the treatment of the patient’s pulmonary squamous cell carcinoma. The latter resulted in the partial remission of pulmonary lesions, as confirmed at follow-up (Figure 3(a–c)).

**Informed consent:** Informed consent has been obtained from this patient included in this study.

## 3 Discussion

Cystic and cavitary lung lesions are commonly encountered in daily clinical practice and often pose a diagnostic challenge for clinicians. The coexistence of a malignancy and a pulmonary infection is not so infrequent, but the diagnostic evaluation of cavitary lung lesions is complex. Multiple cases have been reported in the literature in which cavitary pulmonary lesions appear to host a combination of a malignancy and an infectious pathogen. Cases of concomitant cavitary pulmonary aspergillosis and lung carcinoma have also been reported [6,7,8]. Although the causality between the development of aspergillosis in the presence of a pulmonary malignancy is uncertain, it has been hypothesized that chronic inflammation and tissue scarring may contribute to carcinogenesis and that the immunosuppression associated with cancer and its anticancer therapy may, on the other hand, allow for the development of an infection, such as tuberculosis [9,10]. There is a reduction in cell function in particular natural killer cell in cancer of any type [11]. Tumors have increased secretion of proinflammatory mediators that include interleukin 6 (IL-6) and tumor necrosis factor, which have also been shown [12]. To the best of our knowledge, there have been no cases of metastatic cystic or cavitary lung lesions with fungal infection presented in the literature.

Herein we present a rare case of cystic pulmonary metastases in a patient with tongue squamous cell carcinoma and pulmonary aspergillosis. Both his complex clinical course and radiologic findings posed challenges for the lesions’ differential diagnosis. The conclusive distinction between a neoplasm and a fungal infection is difficult, and a careful observation of the radiographic features is necessary when treating fungus ball-type aspergillosis. The employment of EBUS-TBB is necessary for the diagnosis of concurrent pulmonary aspergillosis and metastatic squamous cell carcinoma. Surgical resection may not be suitable in such metastatic pulmonary malignancy, and the treatment of aspergillosis must be considered previous to the administration of systemic anticancer chemotherapy. Notably, only treatment for tumors is not suitable. Active treatment of aspergillosis with liposoluble antifungal drugs is necessary and successful [13]. This case serves as a reminder to the clinical physician to pay careful attention to cavitary or cystic cancerous lesions with a potential fungal infection.
